# Supplementary material for: A particle-based computational model to analyse remodelling of the red blood cell cytoskeleton during malaria infections
Source: PLoS Comput Biol. 2022 Apr 8;18(4):e1009509. doi: 10.1371/journal.pcbi.1009509 (PMC9020725; doi:10.1371/journal.pcbi.1009509)
Supplement: S1 File — S1 Table. Parameters for the repulsion potentials. The value for spectrin is chosen to match previous simulations in Ref. [36]. S2 Table. Summary of actin dynamics rates. Rates and concentrations relevant for the actin dynamics are collected here. The real value is given with its reference where applicable and the value scaled in order to produce observable actin dynamics within an accessable time period. The scaling factor b was set to 105. S3 Table. Experimental values for KD. Dissociation constants are listed, that have been found by various groups for different protein (fragment) pairs. The three parts of the table show interactions of host cytoskeletal proteins, interactions with KAHRP and interactions with the cytoplasmic domain of PfEMP1 from top to bottom. (ZIP) [file pcbi.1009509.s001.zip › S1_File.pdf]

# Supporting information file for: A particle-based computational model to analyse remodelling of the red blood cell cytoskeleton during malaria infections

Julia Jäger[1,2], Pintu Patra[1,2], Cecilia P. Sanchez[3], Michael Lanzer[3*], Ulrich S. Schwarz[1,2*]

[1]Institute for Theoretical Physics, Heidelberg University, Heidelberg, Germany
[2]BioQuant-Center for Quantitative Biology, Heidelberg University, Heidelberg, Germany
[3]Center of Infectious Diseases, Parasitology, University Hospital Heidelberg, Heidelberg, Germany

* Michael.Lanzer@med.uni-heidelberg.de (ML); * schwarz@thphys.uni-heidelberg.de (USS)

## Model Details

Particle and filament properties are determined through a number of different potentials. For monomers, these are a harmonic repulsion to mimic excluded volume effects and a Lennard-Jones potential:

$$\begin{aligned} V_{\text{repulsion}}(|\vec{r}_1 - \vec{r}_2|) &= \frac{k_{\text{repulsion}}}{2}(|\vec{r}_1 - \vec{r}_2| - (r_{c,1} + r_{c,2}))^2 \quad && \text{if } |\vec{r}_1 - \vec{r}_2| < r_{c,1} + r_{c,2} \\ &= 0 && \text{otherwise,} \end{aligned} \tag{1}$$

with $r_c$ the collision radius corresponding to the particle's extend as given in the main text.

$$\begin{aligned} V_{\text{LJ,trunkated}}(|\vec{r}_1 - \vec{r}_2|) &= V_{\text{LJ}}(|\vec{r}_1 - \vec{r}_2|) - V_{\text{LJ}}(r_c) \quad && \text{if } |\vec{r}_1 - \vec{r}_2| < r_c \\ &= 0 && \text{otherwise,} \end{aligned} \tag{2}$$

using

$$V_{\text{LJ}}(|\vec{r}_1 - \vec{r}_2|) = -\frac{\epsilon}{\left(\frac{\sigma}{r_{\min}}\right)^m - \left(\frac{\sigma}{r_{\min}}\right)^n} \left( \left(\frac{\sigma}{|\vec{r}_1 - \vec{r}_2|}\right)^m - \left(\frac{\sigma}{|\vec{r}_1 - \vec{r}_2|}\right)^n \right), \tag{3}$$

with $r_{\min}$ the position of the potential minimum, $V_{\text{LJ}}(\sigma) = 0$, m and n chosen to be 12 and 6, respectively and $\epsilon$ the depth of the potential such that $V_{\text{LJ}}(r_{\min}) = -\epsilon$.

For filaments there is further a harmonic potential between neighbouring particles and an angle potential between three neighbouring beads to tune the filaments persistence length. These are given by:

$$V_{\text{harmonic}}(|\vec{r}_1 - \vec{r}_2|) = k_{\text{bond}}(|\vec{r}_1 - \vec{r}_2| - (r_{c,1} + r_{c,2}))^2 \tag{4}$$

and

$$V_{\text{angle}}(\theta_{1,2,3}) = k_{\text{angle}}(\theta_{1,2,3} - \pi)^2, \tag{5}$$

with $\theta_{1,2,3}$ the angle formed by the connections between beads one and two and beads two and three. $k_{\text{bond}}$ is chosen the same as $k_{\text{repulsion}}$ such that the shapes of the harmonic curves match. Values for $k_{\text{angle}}$ are given in the main text.

## Determining the Shear Modulus

A shear rate of $3 \cdot 10^5$ s$^{-1}$ is applied over 100 discrete shear steps and an equilibration period of 33.33 ns in-between. The network sheared according to the following formula:

$$x_{\text{new}} = x + y \cdot \gamma, \tag{6}$$

$$y_{\text{new}} = y, \tag{7}$$

where $\gamma = [0.05, 0.1, ..., 1.0]$ and $x_{\text{base}}/y_{\text{base}}$ the x/y-extension of the membrane patch. During the simulation we extract the three dimensional area-averaged virial stress tensor of the system:

$$\tau_{ij} = \frac{1}{x_{\text{base}} \cdot y_{\text{base}}} \sum_i \vec{r}_i \otimes \vec{f}_i, \tag{8}$$

where $\vec{r}_i$ is the position vector of particle $i$ and $\vec{f}_i$ the force on particle $i$. For each shear step the stress is taken as the time average of the during the equilibration period, where the first ten percent of the time steps after the shearing are disregarded.

Assuming linear elasticity theory applies and considering a pure shear experiment in the two dimensional x-y-plane, the strain and stress are related by the modulus $G$ in the following way [1]:

$$\tau = \frac{\tau_{xy}}{2} = G\gamma. \tag{9}$$

## Tables

| $k_{\text{repulsion}}$ in $\frac{\text{kJ}}{\text{mol}\,\text{nm}^2}$ | actin | spectrin | adducin | tropomodulin |
|---|---|---|---|---|
| actin | 10 | 38.11 | 10 | 10 |
| spectrin | | 38.11 | 38.11 | 38.11 |
| adducin | | | 10 | 10 |
| tropomodulin | | | | 10 |

**Table S1. Parameters for the repulsion potentials.** The value for spectrin is chosen to match previous simulations in Ref. [2].

| Parameter | Real Value | Scaled Value | Source |
|---|---|---|---|
| cytosolic G-actin concentration | 0.36 µM | 108396 $\mu m^{-3}$ | Gokhin *et al.* [3] |
| cytosolic tropomodulin concentration | 0.005 µM | 1506 $\mu m^{-3}$ | Gokhin *et al.* [3] |
| cytosolic adducin concentration | not known | 1506 $\mu m^{-3}$ | same as tropomod. |
| **macroscopic association rates** | | | |
| barbed end | 11.6 $\mu M^{-1}\,s^{-1}$ | 3.86 $\mu m^3\,s^{-1}$ | Pollard [4] |
| pointed end | 1.3 $\mu M^{-1}\,s^{-1}$ | 0.434 $\mu m^3\,s^{-1}$ | Pollard [4] |
| adducin | not known | 2.5 $\mu m^3\,s^{-1}$ | match $K_D$ |
| tropomodulin | not known | 1.5 $\mu m^3\,s^{-1}$ | match $K_D$ |
| **microscopic association rates** | | | |
| barbed end | no value | $2.38 \times 10^7\ s^{-1}$ | simulation specific |
| pointed end | no value | $5.3 \times 10^5\ s^{-1}$ | simulation specific |
| adducin | no value | $1.11 \times 10^7\ s^{-1}$ | simulation specific |
| tropomodulin | no value | $7.18 \times 10^6\ s^{-1}$ | simulation specific |
| **dissociation rates** | | | |
| barbed end | 1.4 $s^{-1}$ | $1.4 \times 10^5\ s^{-1}$ | Pollard [4] |
| pointed end | 0.8 $s^{-1}$ | $0.8 \times 10^5\ s^{-1}$ | Pollard [4] |
| adducin | not known | $7.5 \times 10^4\ s^{-1}$ | match $K_D$ |
| tropomodulin (with tropomyosin) | not known | $4.52 \times 10^2\ s^{-1}$ | match $K_D$ |
| tropomodulin (no tropomyosin) | not known | $1.35 \times 10^5\ s^{-1}$ | match $K_D$ |

**Table S2. Summary of actin dynamics rates.** Rates and concentrations relevant for the actin dynamics are collected here. The real value is given with its reference where applicable and the value scaled in order to produce observable actin dynamics within an accessable time period. The scaling factor b was set to $10^5$.

| **Protein 1** | **Protein 2** | $K_D$ **in** µM | **References** |
|---|---|---|---|
| Spectrin | F-actin | 200 | Ohanian *et al.* 1984 [5] |
| Spectrin | protein4.1 | 0.1 | Tyler *et al.* 1980 [6] |
| | | 0.1 | Podgorski *et al.* 1985 [7] |
| | | 2.0 | Eder *et al.* 1986 [8] |
| | | 0.2 | Li *et al.* 2014 [9] |
| | (phosphorylated) | 9.6 | Eder *et al.* 1986 [8] |
| Spectrin | Ankyrin | 0.1 | Tyler *et al.* 1980 [6] |
| | | 0.05 | Bennet *et al.* 1980 [10] |
| Ankyrin | Band3 | 0.01 | Bennett *et al.* 1980 [10] |
| | | 0.01 | Thevenin *et al.* 1990 [11] |
| Spectrin | Spectrin | 2 | Liu *et al.* 1981 [12] |
| Tropomyosin | F-actin | 0.4 | Fowler *et al.* 1984 [13] |
| Adducin | F-actin | 0.1 | Gardner *et al.* 1987 [14] |
| Tropomodulin | F-actin with tropom. | 0.001 | Fowler *et al.* 1990 [15] |
| | F-actin | 0.3 | Weber *et al.* 1999 [16] |
| KAHRP | Ankyrin (MBD) | 1.8 | Magowan *et al.* 2000 [17] |
| KAHRP | Ankyrin (D3) | 1.3 | Magowan *et al.* 2000 [17] |
| KAHRP K1D | Ankyrin (MBD) | 0.038 | Weng *et al.* 2014 [18] |
| KAHRP K1D | Ankyrin (D3) | 0.046 | Weng *et al.* 2014 [18] |
| $VAR_{CD}$ | F-actin | 0.04 | Oh *et al.* 2000 [19] |
| $VAR_{CD}$ | KAHRP | 0.01 | Oh *et al.* 2000 [19] |
| VARC | KAHRP (K1A) | 0.1 | Waller *et al.* 1999 [20] |
| VARC | KAHRP (K2A) | 3.3 | Waller *et al.* 1999 [20] |
| VARC | KAHRP (K2A1) | 32.06 | Ganguly *et al.* 2015 [21] |
| VARC | KAHRP (K3) | 13.0 | Waller *et al.* 1999 [20] |

**Table S3. Experimental values for $K_D$.** Dissociation constants are listed, that have been found by various groups for different protein (fragment) pairs. The three parts of the table show interactions of host cytoskeletal proteins, interactions with KAHRP and interactions with the cytoplasmic domain of PfEMP1 from top to bottom.

## References


1. Landau LD, Lifshitz EM, Kosevich AM, Pitaevskii LP. Theory of Elasticity: Volume 7. Elsevier; 1986.

2. Li J, Lykotrafitis G, Dao M, Suresh S. Cytoskeletal dynamics of human erythrocyte. Proceedings of the National Academy of Sciences. 2007;104(12):4937–4942. doi:10.1073/pnas.0700257104.

3. Gokhin DS, Nowak RB, Khoory JA, Piedra Adl, Ghiran IC, Fowler VM. Dynamic actin filaments control the mechanical behavior of the human red blood cell membrane. Molecular Biology of the Cell. 2015;26(9):1699–1710. doi:10.1091/mbc.E14-12-1583.

4. Pollard TD. Rate constants for the reactions of ATP- and ADP-actin with the ends of actin filaments. The Journal of Cell Biology. 1986;103(6):2747–2754. doi:10.1083/jcb.103.6.2747.

5. Ohanian V, Wolfe LC, John KM, Pinder JC, Lux SE, Gratzer WB. Analysis of the ternary interaction of the red cell membrane skeletal proteins, spectrin, actin, and 4.1. Biochemistry. 1984;23(19):4416–4420. doi:10.1021/bi00314a027.

6. Tyler JM, Reinhardt BN, Branton D. Associations of erythrocyte membrane proteins. Binding of purified bands 2.1 and 4.1 to spectrin. The Journal of Biological Chemistry. 1980;255(14):7034–7039.

7. Podgórski A, Elbaum D. Properties of red cell membrane proteins: mechanism of spectrin and band 4.1 interaction. Biochemistry. 1985;24(27):7871–7876. doi:10.1021/bi00348a004.

8. Eder PS, Soong CJ, Tao M. Phosphorylation reduces the affinity of protein 4.1 for spectrin. Biochemistry. 1986;25(7):1764–1770. doi:10.1021/bi00355a047.

9. Li H, Lykotrafitis G. Erythrocyte membrane model with explicit description of the lipid bilayer and the spectrin network. Biophysical journal. 2014;107(3):642–653.

10. Bennett V, Stenbuck PJ. Association between ankyrin and the cytoplasmic domain of band 3 isolated from the human erythrocyte membrane. The Journal of Biological Chemistry. 1980;255(13):6424–6432.

11. Thevenin BJ, Low PS. Kinetics and regulation of the ankyrin-band 3 interaction of the human red blood cell membrane. The Journal of Biological Chemistry. 1990;265(27):16166–16172.

12. Liu SC, Palek J, Prchal J, Castleberry RP. Altered spectrin dimer-dimer association and instability of erythrocyte membrane skeletons in hereditary pyropoikilocytosis. The Journal of Clinical Investigation. 1981;68(3):597–605. doi:10.1172/JCI110293.

13. Fowler VM, Bennett V. Erythrocyte membrane tropomyosin. Purification and properties. Journal of Biological Chemistry. 1984;259(9):5978–5989.

14. Gardner K, Bennett V. Modulation of spectrin–actin assembly by erythrocyte adducin. Nature. 1987;328(6128):359.

15. Fowler VM. Tropomodulin: a cytoskeletal protein that binds to the end of erythrocyte tropomyosin and inhibits tropomyosin binding to actin. The Journal of Cell Biology. 1990;111(2):471–481. doi:10.1083/jcb.111.2.471.

16. Weber A, Pennise CR, Fowler VM. Tropomodulin Increases the Critical Concentration of Barbed End-capped Actin Filaments by Converting ADP·Pi-actin to ADP-actin at All Pointed Filament Ends. Journal of Biological Chemistry. 1999;274(49):34637–34645. doi:10.1074/jbc.274.49.34637.

17. Magowan C, Nunomura W, Waller KL, Yeung J, Liang J, Van Dort H, et al. Plasmodium falciparum histidine-rich protein 1 associates with the band 3 binding domain of ankyrin in the infected red cell membrane. Biochimica et Biophysica Acta (BBA)-Molecular Basis of Disease. 2000;1502(3):461–470.

18. Weng H, Guo X, Papoin J, Wang J, Coppel R, Mohandas N, et al. Interaction of Plasmodium falciparum knob-associated histidine-rich protein (KAHRP) with erythrocyte ankyrin R is required for its attachment to the erythrocyte membrane. Biochimica et biophysica acta. 2014;1838(1 0 0):185–192. doi:10.1016/j.bbamem.2013.09.014.

19. Oh SS, Voigt S, Fisher D, Scott JY, LeRoy PJ, Derick LH, et al. Plasmodium falciparum erythrocyte membrane protein 1 is anchored to the actin-spectrin junction and knob-associated histidine-rich protein in the erythrocyte skeleton. Molecular and biochemical parasitology. 2000;108(2):237–247.

20. Waller KL, Cooke BM, Nunomura W, Mohandas N, Coppel RL. Mapping the binding domains involved in the interaction between the plasmodium falciparum knob-associated histidine-rich protein (KAHRP) and the cytoadherence ligand P. FalciparumErythrocyte membrane protein 1 (PfEMP1). Journal of Biological Chemistry. 1999;274(34):23808–23813.

21. Ganguly AK, Ranjan P, Kumar A, Bhavesh NS. Dynamic association of PfEMP1 and KAHRP in knobs mediates cytoadherence during Plasmodium invasion. Scientific Reports. 2015;5:8617. doi:10.1038/srep08617.
