# Supplementary figures and images for: A particle-based computational model to analyse remodelling of the red blood cell cytoskeleton during malaria infections
Source: PLoS Comput Biol. 2022 Apr 8;18(4):e1009509. doi: 10.1371/journal.pcbi.1009509 (PMC9020725; doi:10.1371/journal.pcbi.1009509)

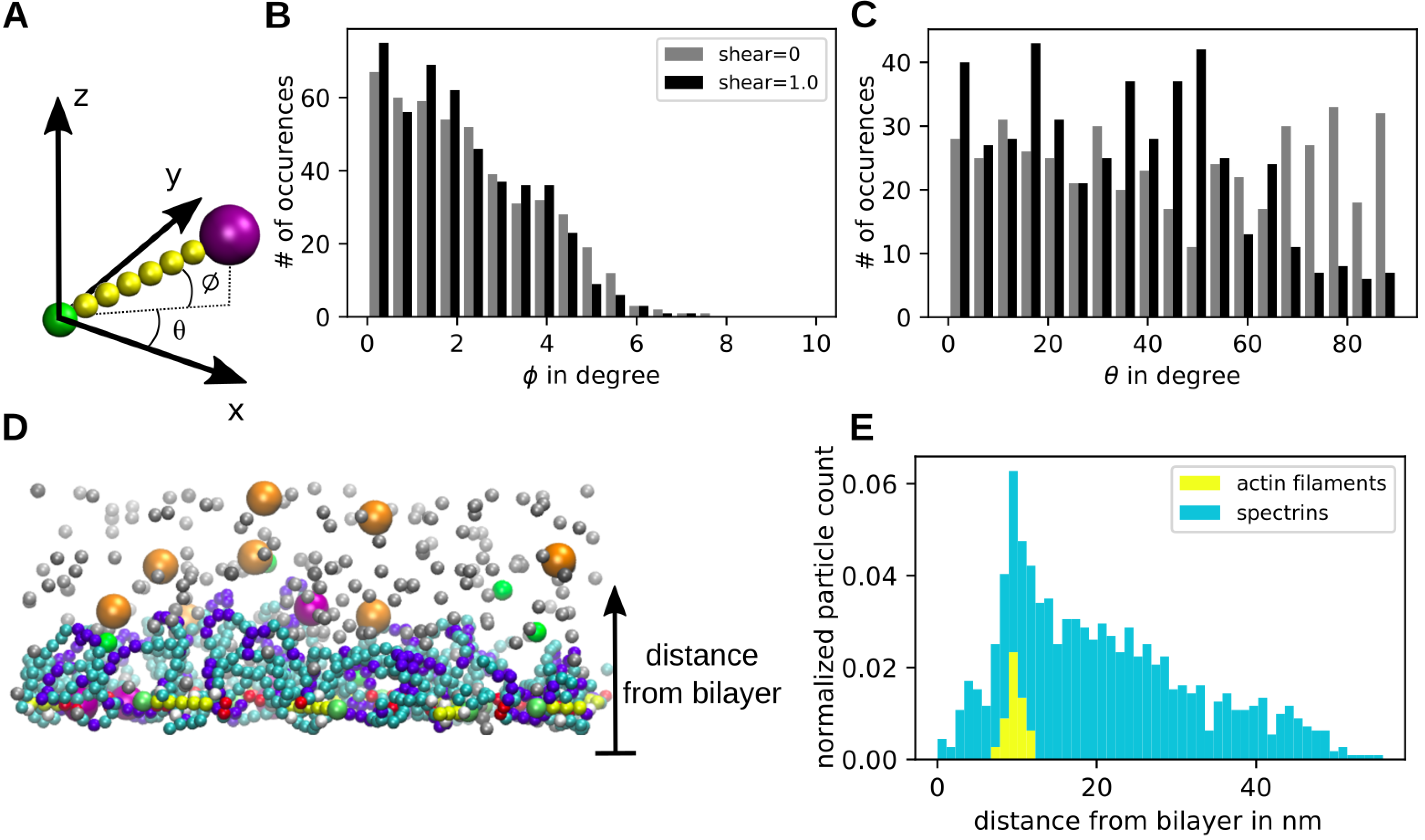

Supplement: S1 Fig — (A) Definition of actin filament angles in space. Both angles can vary between 0 and 90° as the filament polarity does not matter. (B) Distribution of the out of plane angle ϕ at the initial and final time point of the simulation. The data from 10 independent runs of a network with 46 actin filaments each is used. (C) Same as in b but for the orientation within the plane of the bilayer quantified with angle θ. (D) Side view of a simulation snapshot with a confining potential for the actin filaments near the bottom of the simulation box. Actin filaments are shown in yellow and spectrin filaments in cyan and blue. Diffusing monomers are important for the actin polymerisation. (E) Distribution of different particle types given as distance from the bottom simulation boundary mimicking the lipid bilayer. Actin filaments and spectrin filaments are considered separately. (TIF) [file pcbi.1009509.s002.tif]

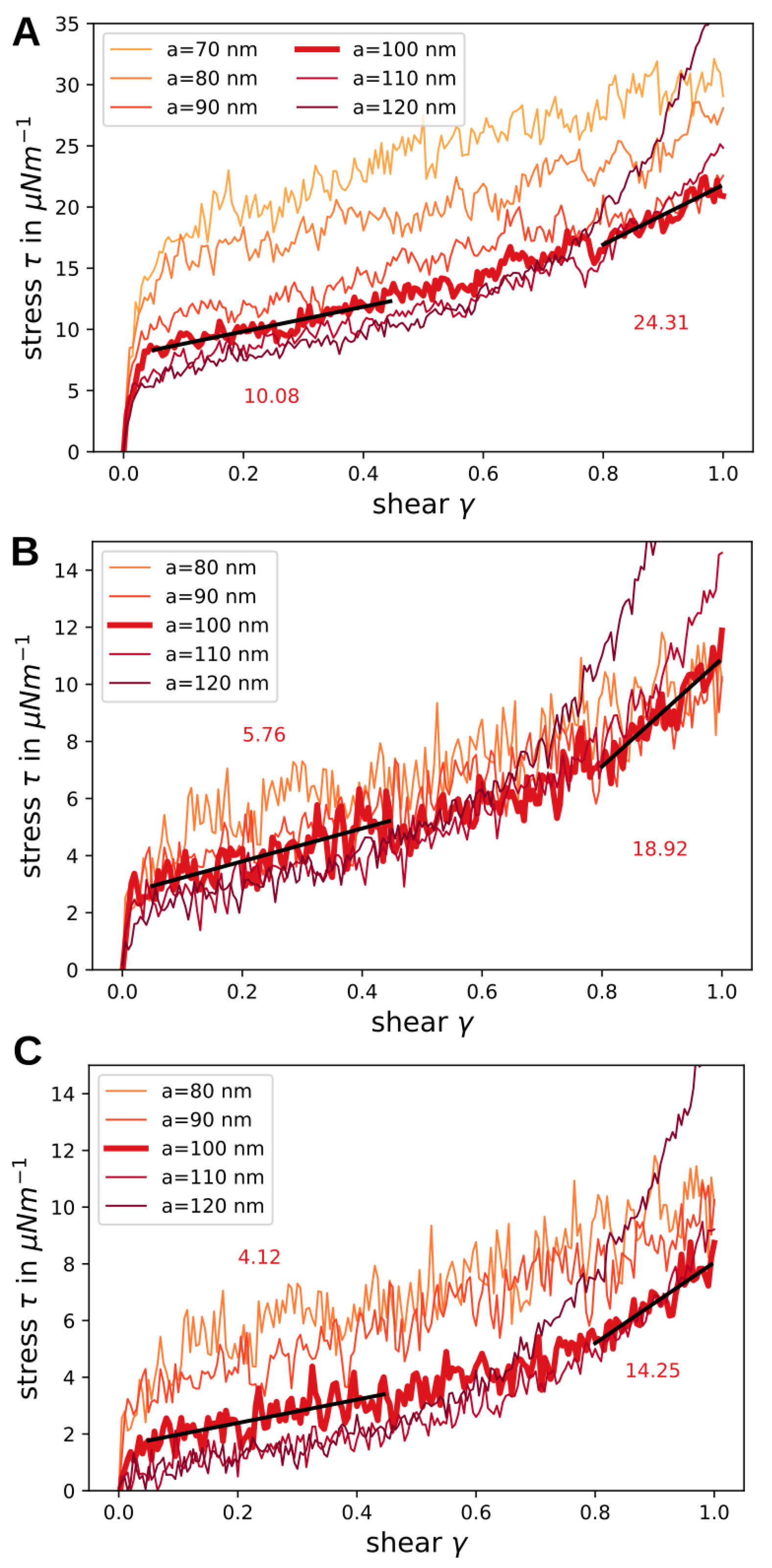

Supplement: S2 Fig — Effect of different shear rates on the stress in the modelled network is shown for different lattice constants. (A) The shear rate was set to 1.5 ⋅ 105 s−1. (B) The shear rate was set to 5.0 ⋅ 104 s−1. (C) The shear rate was set to 1.7 ⋅ 104 s−1. (TIF) [file pcbi.1009509.s003.tif]

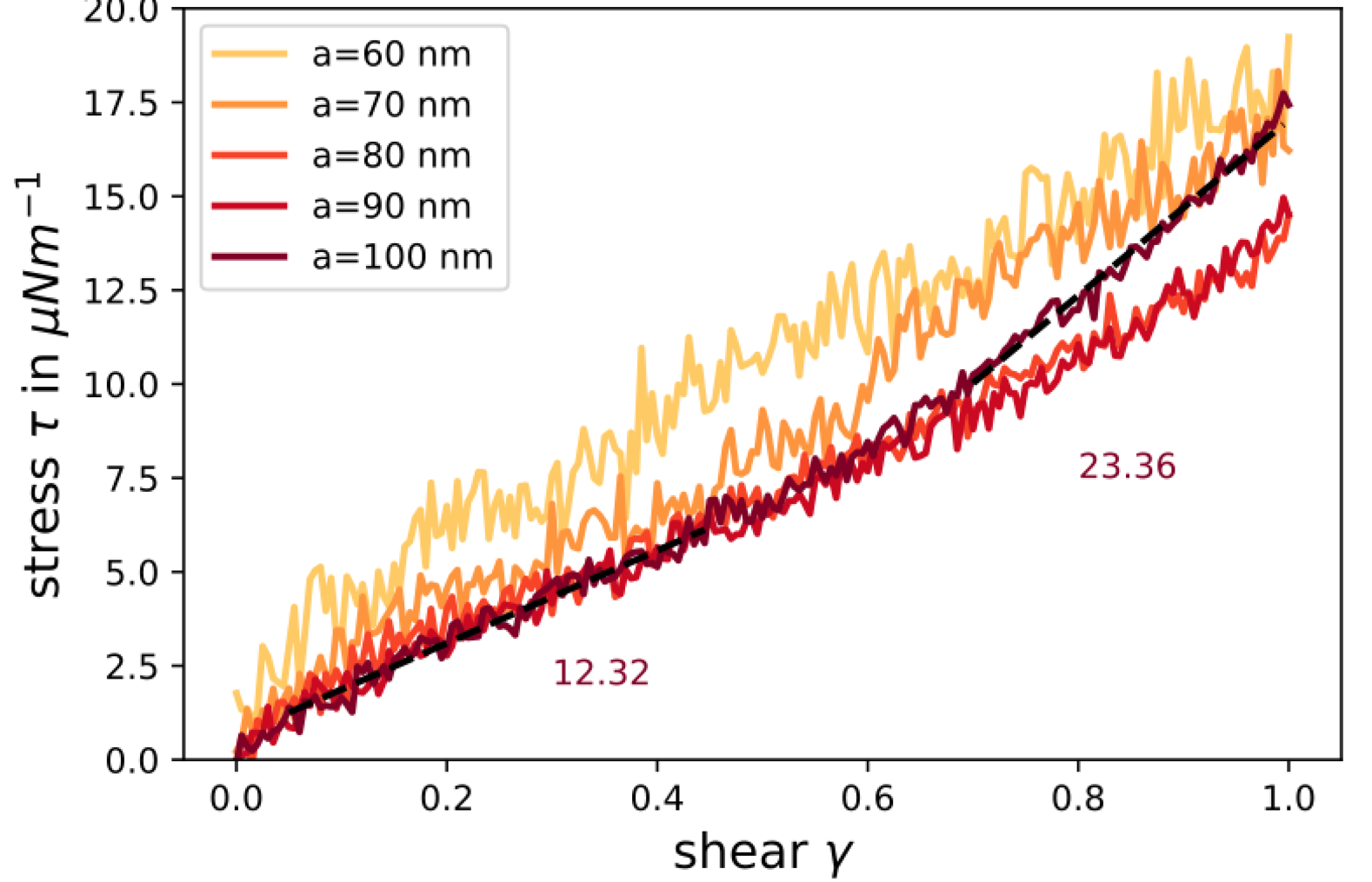

Supplement: S3 Fig — For these shear simulations the position of the actin particles was randomly displaced from their hexagonal lattice site. The extracted stress is shown here for different lattice constants. (TIF) [file pcbi.1009509.s004.tif]

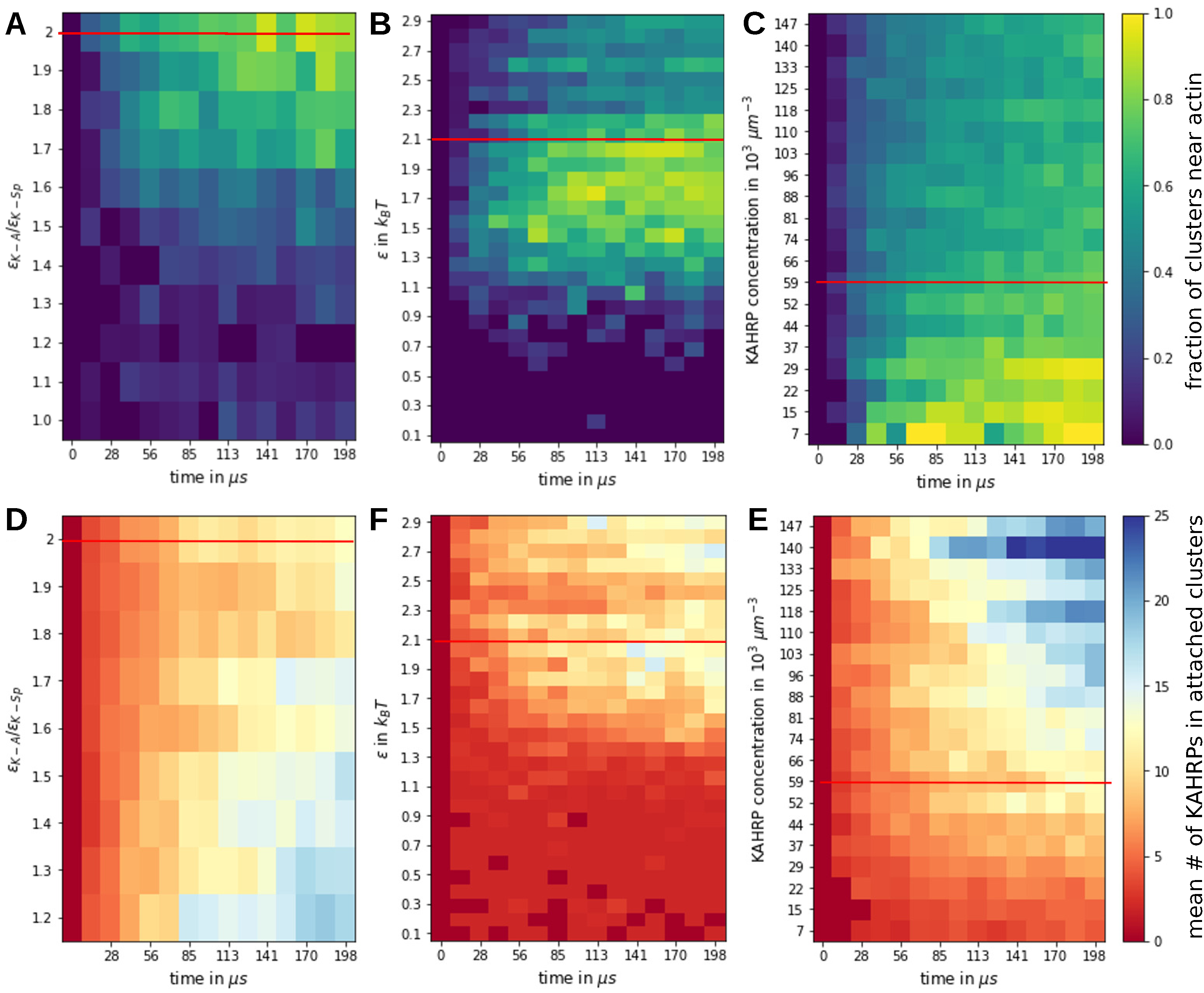

Supplement: S4 Fig — (A-C) The fraction of clusters near actin filaments is plotted over time and distinct conditions as explained hereafter. (D-F) Average size of cytoskeleton attached clusters is plotted for the same conditions. In (A) and (D) the relative strength between the KAHRP-actin and the KAHRP-spectrin interaction is varied. In (B) and (E) the interaction strength is varied and in (D) and (F) the KAHRP concentration. For each of the three columns the other two parameters are kept fixed at the value indicated by the red horizontal line. Each data point corresponds to the average of three simulations. (TIF) [file pcbi.1009509.s005.tif]

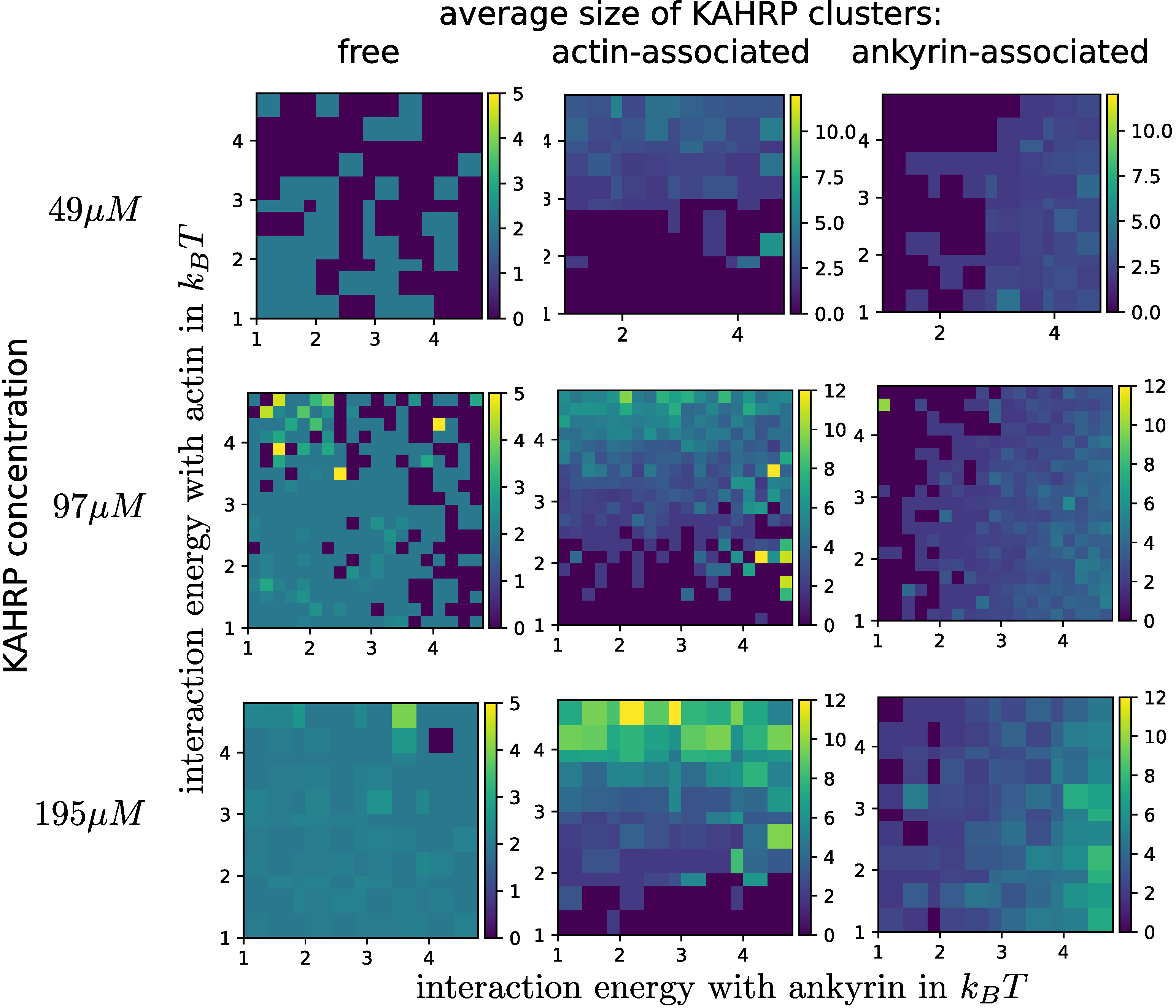

Supplement: S5 Fig — The map shows the average size of different KAHRP clusters, i.e., free, actin-associated, and ankyrin-associated for different binding energy between KAHRP and ankyrin/actin junctions and for different concentrations of KAHRP. (TIF) [file pcbi.1009509.s006.tif]

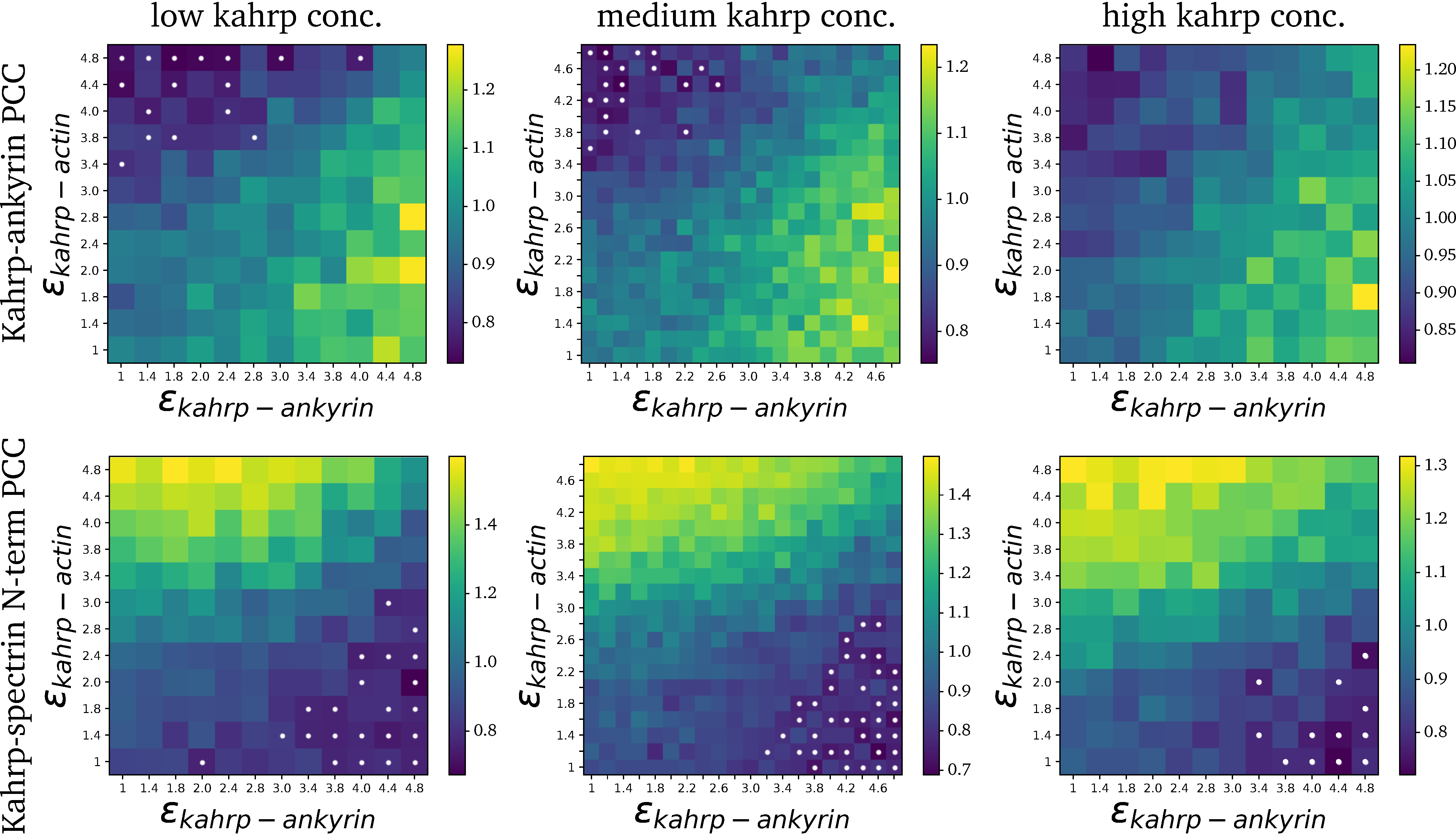

Supplement: S6 Fig — The map shows PCC at zero distance for KAHRP-actin pairs (top panel) and KAHRP-ankyrin pairs (bottom panel) for different KAHRP concentrations shown in S4 Fig. (TIF) [file pcbi.1009509.s007.tif]
