# Supplementary material for: A particle-based computational model to analyse remodelling of the red blood cell cytoskeleton during malaria infections
Source: PLoS Comput Biol. 2022 Apr 8;18(4):e1009509. doi: 10.1371/journal.pcbi.1009509 (PMC9020725; doi:10.1371/journal.pcbi.1009509)
Supplement: Attachment — Submitted filename: Reply.pdf [file pcbi.1009509.s014.pdf]

We thank the three expert reviewers for their comments, which we found to be extremely helpful. Below we respond point-by-point to these comments. We also provide a markup copy of the revised manuscript so that our changes can be tracked easily. Title, abstract and author summary are unchanged, but the text has seen extensive revisions and we have added one new figure (new Fig. 5). Note that the notation of Eq. 1 has been adapted to the original paper on ReaDDy 2 (no change in meaning, but earlier we had used the form of the Langevin equation as given in the ReaDDy 2 tutorial) and that we have changed “Figure” to “Fig” throughout the manuscript to comply with journal style. These changes and also some other small corrected typos have not been marked. We have uploaded representative ReaDDy 2 scripts to GitHub. We hope that after our extensive revisions, this manuscript can now be accepted for publication in PLoS Computational Biology.

Reviewer #1: The authors present a detailed and interesting study of the effects of the malaria proteins on the RBC cytoskeleton shear stiffness and organization. I think this is important, and the tools are appropriate.

We thank reviewer #1 for this positive assessment.

I have some comments that need addressing before publication
1) A few English editing is needed:
Line 13: "the membrane and there incorporation into the existing surface"
Line 39: "finding that malaria parasite mine actin from the junctional complexes, which then is used to"

These wordings have been corrected. In the second case, the whole paragraph has been rewritten (see below).

2) All the proteins denoted in Fig.1 should be explained in the caption.

For Fig. 1A, we now explain both in the caption and in the main text that anion exchanger 1 (AE1, also known as band 3) and glycophorin C (GPC) are the two main transmembrane proteins responsible for the anchoring of the skeleton to the membrane. AE1 binds ankyrin at the ankyrin complexes, and GPC binds to the actin protofilaments at the actin junctional complexes. We also note that band 3 is very abundant in RBCs (1.2 million times according to Lux Blood 2016) because it is contained in both types of complexes and free in the membrane. In our simulations, we harmonically confine these two junctions to the bottom plane of the simulation box which represents the membrane.

The corresponding changes are made in the introduction (lines 35-39) and to the caption of Figure 1.

3) Line 145: "Since the dissociation constant with spectrin repeats is much larger than the one with ankyrin, we neglect the binding to the blue beads and consider binding to ankyrin as binding to the red beads in Figure 2A. Note that we do not model ankyrin explicitly but KAHRP binds directly to the ankyrin binding site on the spectrin molecule."

But this way the KAHRP will bind to spectrin on the simulated membrane, and in reality it can displace the spectrin from its membrane anchoring proteins and therefore remain only attached to the ankyrin/actin on the membrane, and in the process dissociate the spectrin filaments. Why dont they consider this possibility ?

This is an interesting suggestion but to our understanding this is unlikely to happen. Different from what was written in the original submission, KAHRP does not bind to the spectrin binding site of ankyrin, but to its band 3 binding site (Weng BBA 2014). At the same time, it also binds to the alpha-spectrin chain, thus crosslinking ankyrin and spectrin, but a competition with the ankyrin-spectrin binding site has not been reported (Weng BBA 2014). In our imaging experiments (Sanchez Mol Microbiol 2021), we found that during the initial phase of intraerythrocytic parasite development, KAHRP was close to both the C-terminus of beta-spectrin and ankyrin, also suggesting that it does not decouple the spectrin from the ankyrin bridge. We note that such a process should lead to stronger membrane flickering, while experimentally the opposite is the case (Fröhlich Comm Biol 2019), which usually is interpreted as an increase in connection between membrane and skeleton.

We now clarify in the paragraph on KAHRP-binding in the methods section (lines 167-171) that KAHRP binds to ankyrin at the band 3 binding site and not at the spectrin binding site. Because here we identify ankyrin and its binding site on spectrin, this amounts to the red beads in Figure 2A, which are also used to confine the ankyrin complexes to the plane of the membrane. Note that there are two such binding regions for ankyrin on spectrin, each with two spectrin repeats, one left and one right of the tetramerization site, but that for steric reasons ankyrin can only bind on one side (Lux Blood 2015), so we choose one by random in our simulations. This information has been missing before and is now also included (lines 127-131).

4) Why do they only consider thermodynamic equilibrium interactions and no ATP-induced dissociations of the spectrin-actin junction ? or spectrin tetramer dissociations ?

This is a very interesting suggestion. The effect of ATP-depletion on RBCs is a very important subject and it is well known that it strongly decreases the fluctuation amplitude of the membrane (compare e.g. Tuvia PNAS 1997, Gov and Safran BPJ 2005, Park PNAS 2010, Turlier Nat Phys 2016). A common explanation is that ATP leads to dissociation of spectrin from the actin junctions, which then buckle up and give a kick to the membrane. Potential molecular players here are glycophorin C and protein-4.1.

In the case of P. falciparum-infected erythrocytes, it has been shown that ATP is released via parasite-induced new permeation pathways in the red blood cell membrane (Akkaya Pfluegers Arch – Eur J Physiol 2009), but that the parasite maintains the ATP equilibrium by exporting ATP into the erythrocyte cytosol (Choi and Mikkelsen Exp Parasitol 1990). We conclude that the changes in shear modulus for iRBCs are more likely caused by cytoskeletal remodelling as discussed here and less by changes in ATP-concentration.

We now have added a new paragraph at the end of the discussion in which we discuss the potential role of ATP and that its investigation would be a very interesting future avenue of research for iRBCs (lines 636-652). In the same paragraph we now also discuss the role of the membrane (see below).

5) Can the shear response calculated in Fig.4 be compared in more detail to experimental results ?

We first note that our simulation results for the long-term shear response are in very good agreement with published experimental (Suresh Acta Biomaterilia 2005, Park PNAS 2008) and computational results (Li PNAS 2007, Zhang PNAS 2015, Chang PLOS CB 2016, to name a few representative papers that are cited in our work). Our predictions for the short-

term shear response, when actin protofilaments undergo an ordering transition, are new and should now be confirmed by experiments. However, such experiments might be very challenging and had to be designed with this purpose in mind. In particular, the usual tweezer experiments might not be sufficiently sensitive to see this effect. In general, both the exact orientation of the protofilaments and the lateral homogeneity of the actin protofilament arrangements are open questions in the RBC-field (compare the preprint by the Fowler lab on the bioRxiv, doi:10.1101/2021.03.07.434292). As already mentioned in the discussion, there are also the possibilities of discrete spectrin unfolding events and bistable switching of actin protofilaments, which could smear out the predicted effect on a larger length scale. We note in particular that protofilament bistability has been suggested from computer simulations (Zhu Biophys J 2007, Asaro and Zhu 2020) but also awaits experimental confirmation.

The second paragraph of the discussion addresses these issues and in particular points out the experimental challenges in demonstrating our new predictions and the possible corrections arising from spectrin unfolding and protofilament switching, which are not included in our simulations because we focus on questions of malaria infections. We now cite the recent review by Asaro and Zhu 2020 which summarizes some of the open issues in modelling RBCs.

6) The authors consider fully-conneced spectrin networks, but recent work suggests that the spectrin network is not fully connected:Li, He, et al. "How the spleen reshapes and retains young and old red blood cells: A computational investigation." PLOS Computational Biology 17.11 (2021): e1009516.

A large percentage of dissociated filaments in the normal RBC network was also discussed theoretically in:

Gov, N. S., and S. A. Safran. "Red blood cell membrane fluctuations and shape controlled by ATP-induced cytoskeletal defects." Biophysical journal 88.3 (2005): 1859-1874.

We completely agree that these are important aspects of the system and particularly relevant for the questions of malaria infections discussed here. We now have performed additional simulations with non-perfect networks. These new results are now included as Figure 5 in the main text. Simulations were set up with different perturbations to the network. We first addressed the effects of removing spectrin filaments or stretching some of them to higher extensions. In the new Fig. 5C we see that these changes affect the stress at high shear, where the stress is decreased for three spectrins per node and elevated for the long spectrins. We also studied the effect of removing actin nodes (either only the protofilaments or whole hexagonal units). In the new Fig. 5D we see that both has a similar effect and decreases shear stress. Overall one can say that taking away some nodes reduces the stress at all shear strengths. Our result that decreased connectivity decreases the shear modulus is in agreement with the classical work by Gov and Safran on the effect of ATP dissociating the actin-spectrin connections (Biophys J 2005) and the more recent computational work by the Karniadakis group (Li Biophys J 2018, Li PLoS CB 2021) who studied this effect in the context of reticulocyte maturation, which also comes with reduced connectivity. It is interesting to note that senescence (which comes with ATP-depletion), reticulocyte maturation and malaria infections all share the feature that changes occur at the actin junctional complexes that have a strong effect on mechanical quality control in the spleen.

The new Fig. 5 addresses this important point. The paragraph explaining it also now includes the text on Fig. S3, which has not changed (effect of moving the actin junctions). In the

discussion, we now added a new paragraph on iRBC-stiffening (lines 614-635) that also discusses this subject.

7) Fig.5a: please define the KAHRP molecules, what is their color ? provide zoom-in images of the clusters that they make in each of the cases.

The KAHRP-particles are grey. We now explain this in the caption and have added zoom-ins as suggested to old Fig. 5 (now Fig. 6).

8) I dont see that in any of the main results there is a crucial role for the "actin mining" that was promoted in the abstract. Maybe this is not such an important process ?

Actin mining is one of the central processes during a malaria infection as the parasite remodels the host actin to generate long actin filaments required for vesicular transport of parasite encoded immune-variant adhesins and transport pathways to the host cell plasma membrane (Cyrklaff Science 2011 and Nat Comms 2016). Actin mining is addressed in the old Figs. 3, 4 and 5 (now 6) and, in addition, in new Fig. 5 in the revised manuscript. Throughout the text we now have added comments how important actin mining is, and we have added a whole new paragraph to the discussion to discuss the relation between actin mining and stiffening (lines 610-630). Here we deal with a multifaceted process with several effects: actin mining takes away actin filament length and this decreases stress as shown in Fig. 4I. Then it generates holes that also decrease stress as shown in Fig. 5D. This agrees with previous theory (Gov and Safran Biophys J 2005) and simulation (Li Biophys J 2018, Li PLoS CB 2021). However, it also makes space for longer spectrin filaments and knobs (clusters of KAHRP), which increase stress again, as shown in Figs. 5C and 6C. Overall the last two effects dominate and lead to shear stiffening.

9) In the context of Fig.6: They also do not consider that as time progresses there can be an accumulation of changes to the spectrin network that will also affect the localization of the KAHRP molecules, such as more spectrin-actin dissociations which will make more actin available for KAHRP binding.

We agree, but the main idea is to focus on KAHRP as the causative agent of the remodelling, and not to consider other changes to which KAHRP might respond. In fact there are other parasite-secreted proteins like MESA that might cause such changes at the actin junctional complexes. Our work is strongly informed by the experimental results that we and others have obtained before for KAHRP. In our previous work using super-resolution microscopy, we showed that the KAHRP-induced knobs assemble at remnant actin junctions (Sanchez et al. Mol Microbiol 2021). Remnant actin junctions means that the typical actin junctional complex is no longer maintained in knobs and is replaced by a new, hitherto not yet fully understood arrangement that involves a KAHRP-containing spiral structure. The KAHRP contained in the knob spiral colocalizes with the N-terminus of ß-spectrin, protein 4.1R and actin, but not the actin capping factors adducin and tropomodulin. On the basis of these findings, we proposed the model that the KAHRP-containing spiral replaces the protofilaments in their function to connect the spectrin filaments and that the observed association with actin reflects the long actin filaments that extend from the spiral towards the parasite (respectively parasite induced membrane profiles, termed Maurer’s clefts) and which are required for antegrade vesicular protein transport towards the red blood cell plasma membrane.

In the discussion (lines 551-554), we now have added a clear statement that a full simulation of the dynamical remodelling process is not possible at the current stage due to missing experimental information and that our simulation study is meant to quantitatively highlight the role of actin and KAHRP in this process.

Reviewer #2: The authors developed a sophisticated red cell membrane model to study its dynamics in malaria-infected cases with KAHRP proteins. It is an interesting study, which might be helpful for understanding better the red cell mechanics and malaria. Since KAHRPs interact with actin,s the polymerization process might be important, which is captured in this study for the first time.

We thank reviewer #2 for this positive assessment.

The major concern is that some basic elements might not be right in this model, in spite of many advanced features, such as actin polymerization.

When simulating a complex biological process, such as the re-organization of the red blood cell membrane skeleton by the malaria parasite, there is always concern that, because of gaps in our current knowledge and the required abstraction, the model illuminates only a small part of the whole picture. We are aware of these limitations and have extended the discussion to emphasize this point. Irrespective of possible shortcomings of our simulation, we revealed several novel details. This includes the details of the interaction of KAHRP with ankryrin, spectrin and actin, and the impact this has on the stiffness of the membrane skeleton, which is the main determinant of RBC-quality control in the spleen. The discussion now contains a clear statement (lines 551-554) that a full simulation is not yet possible due to missing information. The discussion has been extended in several ways to give a more comprehensive overview of open issues.

Here are the major questions:

1. The spectrin tetramer have two strands, but it is modeled as one. Even ankyrins will be doubled if it is modeled as two than one. Is it possible to model it as two strands to see the difference? There seems no particular technical challenges to do so, especially considering that more complicated features such as polymerization are modeled.

During assembly of the dimer, the alpha and beta chains of spectrin are known to zip up from the actin-binding tail that is held together by electrostatic interactions (compare Fig. 2A). Then two dimers associate head-to-head to form a tetramer which is believed to stay together as single strand for most of the time. It is also believed that the two chains might slide relatively to each other (Lux Blood 2015). It is not clear how often the triple helical repeats unfold, but certainly these events would be less discrete in the physiological context than in single molecule experiments (Rief JMB 1999). If a study is not focused on the exact function of spectrin, it is the standard approach in RBC computer simulations to simulate only a single strand, with the 39 beads representing the triple helical repeats along its 200 nm length (Chang et al., PLoS Comput Biol 2016).

We now clarify in lines 113-120 that the spectrin tetramer is believed to stay together as one strand for most of the time and that this is the standard approach for RBC-modelling. The

reason why here we model actin in more detail than spectrin is of course our interest in malaria infections, which lead to actin mining and knob formation.

2. Many recent studies showed that spectrins might not behave as random coils at all, such as the Chinese Finger Trap Model by McKnight group, since many EM images showed the spectrin maintains straight-line shape rather than random-coil shape in the native state. Any discussion on this? If the spectrin model is wrong, no matter how many advanced features about actin are added, the model might not be realistic.

The Chinese Finger Trap model is an interesting suggestion in the long-standing discussion on the exact configurations of the spectrin chains. As explained above, however, it is the standard approach for RBC-simulations to use the WLC-model for spectrin. We note that this model predicts a typical junction distance of 70 nm and a typical layer thickness of 50 nm, in good agreement with experimental results (Pan Cell Reports 2018 and Sanchez Mol Microbiol 2021 for distance and Heinrich Biophys J 2001 for thickness). We also note that imaging the RBC-skeleton at molecular detail is still a huge challenge because most microscopy preparations lead to some degree of contraction or stretching in the network. In particular, early EM images gave much too small numbers for junction distance. It is expected that future advances will be able to clarify these important questions, but they are not the focus of our work, which is on malaria infections. Because we do not include the weak (blue) binding sites along the spectrin, but only consider the strong (red) binding sites (compare Fig. 2), the exact conformation of the spectrin chains does not matter so much for the predictions on KAHRP-clustering. In the context of Brownian dynamics, it is more important to get reasonable statistics of the chain conformations, such that all possible conformations for binding are indeed explored.

On lines 262-265, in the paragraph where we show that our simulations give very good WLC-statistics, we now explain that our focus is the statistics of the spectrin chains and not on their molecular details. Here we cite the Chinese finger trap paper as interesting alternative if one wanted to focus more on spectrin. We now also cite this paper when we introduce spectrin on line 113, because it nicely explains its molecular structure, including molecular renderings of the triple-helix repeats and their linkers which essentially make spectrin an effective WLC (Fig. 1C in the Brown PLoS CB 2015 paper).

3. One major assumption is that actins constantly polymerize in red cells, as Fowler's group showed so. But it is still a controversial topic. At least, the authors should discuss why they think actin polymerize in red cells with these capping proteins, because for a long time, actin filaments in red cells are assumed to have constant lengths. A discussion of Fowler group's results and alternative opinions might be helpful.

We first note that the early work by Fowler was actually motivated by our own work (Cyrklaff Science 2011) in which it was shown by EM tomography that long filaments are formed from host actin during a malaria infection at the expense of the protofilaments that are mined from the junctional complexes during formation of the long actin filaments. These parasite induced long actin filaments extend from the knobs towards parasite-induced membrane profiles, termed Maurer's clefts, and are required for vesicular transport of parasite encoded proteins to the erythrocyte membrane (Cyrklaff Science 2011).The conclusion of Fowler that not all actin protofilaments are capped and that the equilibrium between monomeric and filamentous actin can be shifted by small inhibitors like LatA or Jasp has recently been confirmed by the super-resolution microscopy study by Pan who also concluded that not all junctions are fully occupied by capping proteins. In our own recent

study (Sanchez Mol Microbiol 2021), we demonstrated by super-resolution microscopy that the actin that is associated with the knob-forming KAHRP protein is uncapped as it does not co-localize with adducing or tropomodulin. We do not know yet what the underlying molecular mechanisms are and hope to clarify this experimentally in the years to come.

In the introduction (lines 49-51) and discussion (line 632), we now state clearly that the underlying mechanisms for uncapping are not known yet. Throughout the manuscript, we now describe better the essential role of actin mining.

4. Similarly, how these polymerization constants are determined in red blood cells? Table S2 show concentrations of G actin, its two capping proteins (adducin and tropomodulin) in red cell from Gokhin et al.. The authors can search the proteomic data of red cells published recently by Gautier et al. to see whether their assumed concentrations are reasonable. In Table S2, it seems the scaled values of rates are obtained to match K_D. Which K_D? What are references for these K_D?

Although it is not known how exactly the actin protofilaments are formed during reticulocyte maturation, it is clear that their length is not determined only by the capping proteins, but that also tropomyosin plays an important role. It also is an important part of our simulations. Polymerization stops at the length of tropomyosin because tropomodulin associates to the pointed end of actin filaments better in presence of tropomyosin (K_D < 1 nM) (Fowler J Cell Biol 1990). In the absence of tropomyosin the affinity is much smaller (K_D ≈ 0.3 μ M) (Weber J Biol Chem 1999). Adducin (K_D ≈ 100 nM) caps the barbed end of actin filaments and recruits additional spectrin filaments to the junctional complex (Gardner and Vann Bennett, Nature 1987).

Regarding the proteomics data, both the paper by Gautier in Blood Advances 2018 and the one by Bryk and Wisniewski in Journal of Proteome Research 2017 report similar values for the main components of RBCs as the Lux review in Blood 2015. For actin, the concentration is around 10 uM, with 96% F-actin and 4% G-actin. A simple calculation shows that this amount of F-actin leads to the 35.000 actin junctional complexes mentioned in our paper (given a surface area of 140 um^2 and a volume of 100 um^3 for the RBC). The amount of G-actin corresponds well to the 0.36 uM value reported by the Fowler group and mentioned in our Table S2. This also sets the scale for the values used in Fig. 3E and F (after appropriate rescaling for the simulations).

We now have added a new Table S3 with all K_D values and mention it when we mention Table S2 with concentrations and rates (line 196). Because we do not use these data here, we decided not to mention or cite the proteome papers.

5. Since the spectrins are modeled as entropic springs, sufficient sampling is the key to capture its free energy. Although Fig. 3B showed it matches with some theories, is it possible to provide some data to show the results are converged with sufficient sampling (like longer simulations) for both single spectrin and spectrin networks? A brief description of the difference between Flory theory and WLC/FJC models will be helpful.

We agree that Fig. 3B has not been explained sufficiently well. Its main purpose is to demonstrate that our simulations works well and that different polymer models can be implemented in ReaDDy 2, although in the end we use the WLC for the spectrin and actin.

We now have rewritten the first two paragraphs of the results section (lines 236-265) to clarify that the simulations from Fig. 3 are test cases for our implementation and nicely confirm that our WLC-model for spectrin works and gives reasonable values for junction-junction distances and network thickness.

6. The lipid bilayer membrane may play an important role, but it is assumed to a flat plane. Will the flexibility and fluctuations of the lipid bilayer impact the process simulated?

Here we only simulated a finite patch of skeleton and not a whole cell because we focus on molecular details in the cytoskeleton. Earlier particle-based simulations of the RBC-cytoskeleton used very similar system sizes (in particular the work by the Karniadakis group). We also note that membrane fluctuations are expected to be important on larger scales and decrease during infection due to increased connections to the skeleton. We have published before on the dynamics of membrane fluctuations during a malaria infection (Fröhlich Comm Biology 2019), where we used a continuum theory, but here we focus on the changes in the skeleton and use a particle-based approach. In the future, these two lines of research might be brought together, e.g. using the multiscale two-compartment model of the Karniadakis group (Chang PLoS Comp Biol 2016). We also note that ReaDDy 2 does not include any features yet to simulated membranes explicitly.

In the methods section, we now explain in more detail that we simulate only a small patch of membrane and therefore neglect membrane fluctuations (lines 121-123). In the discussion, we have added a whole new paragraph discussing membrane fluctuations (lines 636-652).

7. In addition, using harmonic constraint to fix all particles, including spectrin and actins in 4nm thick plane 10 nm above the bilayer seems very artificially. What are the physical justifications? If they are confined in 4 nm plane 10 nm above, how these thickness of the cytoskeleton (54.21 nm) are estimated later on? Did I misunderstand something?

Only actin and spectrin endbeads and ankyrin attachment sites are confined to the plane as these will be anchored to the membrane by transmembrane proteins. The thickness is the average of the lowest and highest spectrin particles over time. The chosen dimensions correspond to typical dimensions of the relevant transmembrane proteins.

The justification and implementation for the confinement is now explained in more details on lines 126-131.

8. If keeping decreasing the shear rate, will the shear modulus curve converge? The shear rate effect might be artificial, since the hydrodynamic effect is not captured accurately in Brownian dynamics. In addition, what are the major sources of dissipation if it is rate-dependent? Is it from spectrin? But spectrin does not have any dissipation term in the current model. Maybe from the lipid bilayer, but it is not modeled.

The time scale of our particle-based simulations is set by the diffusion constants tabulated in Table 1. Hydrodynamic flows are expected to play only a minor role in the simulated situation, but certainly they would become very important for whole cell modelling, e.g. for flow through the IESs in the spleen. Note that hydrodynamic interactions as well as membrane processes such as flickering or budding cannot not be modeled in ReaDDy 2, which otherwise is a very versatile simulation framework. In our work, hydrodynamics enters only indirectly through the use of the Stokes-Einstein relation from Eq. 2. The shear rate used throughout the manuscript is the standard choice in this field. The smaller shear rates are

included only for completeness. We note that the two smaller shear rates from Fig. S2B and C give very similar results, so they might already be very close to the equilibrium result and seem to converge. This is also suggested by the fact that the jump related to actin reorientation becomes smaller. Going even slower is not possible due to compute time restrictions, but we expect that this would not change the results much anymore.

We now explain on line 323 that our chosen shear rates are standard choices.

9. In Figure 4, is it possible to plot shear modulus as a function of shear?

This would require some kind of averaging of the noisy simulation data. It is common practise to use an average slope to extract a typical value as done here. In general, in the manuscript we discuss both, the shear modulus (as the local slope) and the overall stress levels.

10. It is good to compare the shear responses between simplified model and the full model in Figure 4. But is it possible to plot the case with actin polymerization versus the case without it? Will actin polymerization change the behavior?

This is mostly addressed in Fig. 4F. On the timescale of the shear, not much polymerization is occuring, therefore we sheared networks of different polymerization stages as measured by N_av, the average actin filament length. The actin dynamics shows in the rather dynamic nature of these curves.

To make this clearer the reference case with 6 actin subunits is now plotted in black color in Fig. 4F.

11. Considering the binding between actin, spectrin, and KAHRP can be very helpful for understanding the mechanisms, but it is known the spectrin tetramer can dissociate into dimers? Will modeling this dissociation change the picture? The rate constants were published before for dimer association.

It is known that spectrin can exist in different oligomerization states, but a role of dimers has to our knowledge not yet been described. The more common process seems to be the association of two tetramers into an octamer, which has been seen in EM images. Lacking detailed data, we do not consider neither process, but in new Fig. 5 we now consider the effect if the spectrin tetramer has to span larger distances.

12. How exactly does the spectrin bind to the polymerized actin filament is not clearly described? It is known that spectrin bind to specific locations of the actin filament. How is this achieved in the algorithm? Will the spectrin only bind to certain bead in the actin? Do we know the binding constants between actin and beta spectrin CH end? How to prevent multiple spectrins bind to the same site of the actin if LJ potential is used? Figure 2CD show the LJ potential between actin and spectrin has an equilibrium length of 8nm, any physical justification? Spectrin-spectrin bond is 12 nm in Fig. 2C, but one spectrin repeat is only 5.2 nm as shown in Fig. 2A. Is this a mistake?

Our simulation is the first one that models spectrin-binding to explicitly resolved actin protofilaments. ReaDDy 2 is ideal to simulate this situation because it allows to simulate “topologies”, including the polymers we need. Using LJ-potentials is the usual approach in this context. The exact binding sites are not modelled, however, we chose a repulsive

interaction between spectrin end beads such that each actin bead fits one spectin bead at each side. Thus the connectivity of six spectrin emerges from the typical size of the actin protofilament and changes if the actin protofilament changes length. The equilibrium length of the bonds results from the size of the actin and spectrin monomers.

Thanks for spotting the mistake in Figure 2C. The radius of a spectrin bead is 2.63 nm and hence the bond has a length of 5.26 nm. This has been corrected. Throughout the manuscript we now explain better how our procedures are shaped by the capabilities provided by ReaDDy 2.

13. What are the grey particles in the simulations? Are they G actins? Will G actin be able to nucleate by itself? Any treadmilling behavior?

The grey particles are G-actin in Fig. 2 and KAHRP-molecules in Fig. 6. We now explain this in the corresponding captions.

Treadmilling is an intriguing possibility, especially now that the Fowler group has shown that the free actin monomer concentration is 0.36 uM (which is also the reference concentration used in our simulations, compare Fig. 3) and therefore exactly between the critical concentrations for pointed and barbed ends. However, the existence of the capping proteins and in particular of tropomyosin as well as the many other interactions speak against this possibility. In our simulations, we did not observe treadmilling.

On lines 303-305 we now discuss the actin concentration and that we did not observe treadmilling.

14. The actin polymerization modeling might be related to the work by Fowler group, but Fowler group also believe the myosin plays a major role in red cell mechanics. Will it be difficult to consider myosin in the current model or any discussion on why it is not important in the current study?

Indeed the Fowler group has shown that RBCs contain around 6.200 non-muscle myosin II molecules (corresponding to around 200 minifilaments, each 300 nm large) and that cell shape depends on myosin II contractility. However, to our knowledge this work has not yet been confirmed by other groups and it is very hard to understand how exactly the myosin II could operate on the short, disordered and strongly covered actin protofilaments. We think that this question has to be clarified before one can start including it here. It is unlikely that this is important in our context of malaria infections.

Similar as for the actin treadmilling, we prefer not to comment on this open issue, which is unlikely to be important in our context of malaria infections.

15. It’s claimed that the interaction between KAHRP and ankyrin/actin are more important than its interaction with spectrin. Any data to support this claim?

This is explained after line 162. The K_D for binding to the spectrin sites depicted in blue is 28 times higher than the K_D for binding with the ankyrin binding side depicted in red. When binding ankyrin, KAHRP seems to also bind spectrin, thus crosslinking the two, but not at the ankyrin-spectrin binding site (Weng BBA 2014). As explained above and now in more detail in the main text, this is our justification to identify the ankyrin binding site with the KAHRP binding site in our simulations.

16. 140 x242:48 x100 nm seems too small comparing to existing similar studies. A representative volume element needs to include sufficient network to get reasonable spatial averaging, unless the authors can show when they make the patch larger it won't impact the result. The authors used 9 repeats patch later on. Are these small and big patches giving the same results for stress and shear modulus?

We would like to clarify that all shearing simulations were done with larger patches. Only for the KAHRP assembly simulations we took the small patch, as there are a lot more particles in the simulation and the assembly process takes very long such that we would not have been able to observe it for larger patches. However, for shearing the KAHRP membrane patches, we repeated the initially periodic small patch, such that the shearing curve could be recorded. For the small patch the curve is too noisy to be useful for extracting a shear modulus. The reason for this is that in the formula for calculating the stress, a spatial average is essential which is not given for the small patch, as anticipated by this reviewer. Note that in general our system sizes are similar to the ones used before by others.

17. Figure 2D shows the initial configuration of the network. Are all spectrins with the same initial length 'a' in Fig. 2D? It seems unlikely due to the PBC condition and random orientation of actins. How are the spectrin initial length determined if they are not the same? Fig. 2D also shows the actin consistent of 6 beads, but the actual actin filament has about 13 monomers. If one beads represents 2 monomers, how the rate of polymerization changes, since I believe the rates are obtained between monomers?

For the computer simulations, we prepared initial conditions as described. First the protofilaments are distributed with a distance a and then their orientations are set randomly. Then the spectrin connections are established to get a connected network and finally the network is relaxed before the production run starts.

The questions regarding the actin protofilaments are intriguing. We first note that the polymerization rate should not depend on whether we model it as single or double strand, because to our knowledge only one binding interface exists at the respective end. Also the space available for spectrin binding through LJ-potential would not change. However, the chiral twist of the double strand is a feature that might be very important but which is not modelled here. It is a long-standing riddle in RBC-biophysics that the actin-spectrin binding sites are distributed along a helix, while the spectrin forms essentially a two-dimensional layer (Lux Blood 2015, Asaro and Zhu 2020). It has been suggested from computer simulations that this should lead to bistability in protofilament conformation (Zhu Biophys J 2007, Asaro and Zhu 2020), but this predictions still awaits experimental confirmation.

In the discussion, we now cite the recent review by Asaro and Thu on open issues in RBC-biophysics and also give a more comprehensive list of open issues that should be addressed in future work, including the bistable actin protofilaments.

18. How high is the temperature in these simulations? Does the change in temperature has any effect on results or simulations process, room vs body?

We used the standard choice of ReaDDy (293 K) and no other temperatures were tested. This choice went into the values of D given in Tab. 1. We do not think that temperature is such an interesting parameter here since the effect of temperature on binding constant has been described in detail elsewhere. We cannot exclude that temperature might play a role in the

context of the recurrent fevers coming with malaria, but for most of the time of the infectious cycle it should be constant.

19. There should be bonded co^nnection between actins and the lipid bilayer through glycophorin C, p55, protein 4.1 et al. How will this impact the actin dynamics? Will it be possible to include in the model?

These are important questions, but not the focus of this work, which is on malaria-infections. The effect of the membrane is included in terms of a slower diffusion in the membrane plane and the confinement next to the membrane, but otherwise our focus is on the cytoskeleton. This is now made clear at several places in the revised main text.

20. Finally, the study of the effect of KAHRP on mechanical properties is interesting, but what are the physical mechanisms? After KAHRP binding to the actin or ankyrin, will it prevent other proteins, such as spectrin, to bind? Or will it weaken or strengthen these existing binding? Or it does not change these, but the mass or diffusivity? In these discussions, the authors talked about ‘increased initial step’ or ‘increased jump’, are they step or jump of stress? At this scale, viscous force is much larger than inertial force. Does this mean the cluster and the larger junction has a larger diffusivity, which lead to altered stress? In summary, it is unclear what are the major changes in terms of components and interactions between them caused by KAHRP.

These are intriguing questions some of which have to be left to future advances. Let us first note that KAHRP binds to spectrin mainly by binding to ankyrin (Wang BBA 2013), so it is expected to crosslink rather than dissolve the spectrin-ankyrin interaction. We experimentally found that KAHRP relocalizes to the actin junctions in later parasite stages (Sanchez Mol Microbiol 2021), and here we show that this might result from affinity changes. We believe that such affinity changes result from phosphorylation events, which however have to be identified first experimentally before they can be used for modelling. The end point of this development however is clear, it is the establishment of the knob structures with the spiral scaffold underneath and the adhesion receptors anchored in the plasma membrane. We already know that around 60 KAHRP molecules (Sanchez Mol Microbiol 2021) and 3-4 PfEMP-1 receptors (Sanchez Comms Biol 2019) exist in each of the thousands of knobs. If we understand better how these knobs are formed dynamically, then this might also help to develop therapies against malaria infections. We also think that future work should develop spatial models for the knobs, including the spiral scaffold.

In the discussion, we now have added a new paragraph summarizing our results and conclusions regarding actin and KAHRP (614-635). There we also mention that KAHRP phosphorylation is the next frontier that should be tackled in experiments, because this should lead to the affinity changes which here we show to be key.

Reviewer #3: In this paper, the authors present a detailed model for the RBC cytoskeleton using Brownian Dynamics (using a software package called ReADDY). They focus on the binding of KHARP, a protein that is associated with the malaria parasite, Plasmodium falciparum. They go on to describe a detailed model of the RBC cytoskeleton and the extract its shear modulus under different conditions. The work is thorough and detailed.

We thank reviewer #3 for this positive assessment.

Since the code was not publicly shared, I cannot comment on whether the results are reproducible.

Our code are python scripts for ReaDDy 2 and we now have uploaded three representative scripts to GitHub (https://github.com/usschwarz/networks).

Given the results, I have the following comments

1. How is the membrane represented in this system? Given that the RBC cytoskeleton is closely associated with the membrane, is the membrane even represented in this system? The knobs of KHARP are transmembrane as described in Figure 2. Without any discussion of this, it is hard to evaluate how the results make sense. At the very minimum, the authors should discuss this omission and how it may impact their results.

The lipid bilayer itself is not included in this model because we simulate only a small patch of the skeleton. Including the membrane would be required for a whole-cell model, but then we had to adopt a multiscale strategy as demonstrated by the Karniadakis group (Chang PLoS Comp Biol 2016). In fact we have published on the role of the membrane before during malaria infections using the established concept of the Helfrich bending Hamiltonian (Fröhlich Comm Biol 2019); for the small patch simulated here in large molecular detail, however, we would not be able to capture the whole fluctuation spectrum. In our context, the main function of the membrane is to pin the skeleton to the plane at selected binding sites and to allow for lateral movement due to its fluidity; both features are represented here.

It is correct that the knobs are transmembrane, but here we focus on KAHRP-clustering. The current view is that KAHRP sits on the cytosolic side of the membrane, where it forms the knobs. The knobs serve as a platform on which parasite encoded adhesins, such as PfEMP1 (a family of immune variant trans-membrane proteins), are presented and anchored to the membrane skeleton, such that PfEMP1 can transmit forces through the membrane. KAHRP reaches the membrane first and PfEMP1 is most probably added later to the knobs. (Wickham, The EMBO Journal Vol. 20 No. 20 pp. 5636±5649, 2001). Therefore the transmembrane part of the knobs seems not to play a dominant role for the organization of the knobs.

We now explain at several places in the manuscript that our intention is not to simulate the membrane, see the responses to the other reviewers above. Here it is only used as pinning points to the two types of junctions. In the discussion, we now added a whole new paragraph on membrane fluctuations, including active fluctuations driven by ATP.

2. There are quantitative measurements of RBC cytoskeleton arrangements in two papers the authors cite (ref 17 and 18). Also, ref 17 and 18 contradict each other. So how do the authors results compare against these references? A quantitative comparison is possible with the data the authors have but has not been included. I believe it is a missed opportunity.

We first note that Ref. 18 is still a preprint and we cite it here mainly to show that the field is very dynamic and that open issues exist. It is true that Ref. 17 (Pan Cell Reports2018) seems to show a relatively ordered network, different from Ref. 18, but we note that it also demonstrated that not all actin junctions are capped, thereby already revealing some kind of irregularity. Here however we focus on malaria-infections and not so much on healthy RBCs. For iRBCs, it is well known from AFM-experiments that the spectrin network changes a lot, with holes opening due to actin mining and later densifying around the growing knobs (Shi

PLoS One 2013), and this is exactly the situation addressed here. As emphasized throughout the manuscript, our starting point are mainly own results obtained from super-resolution microscopy for KAHRP relocalization (Sanchez Mol Microbiol 2021). As written above, we hesitate to comment on open issues for healthy RBCs that do not directly affect our work on malaria.

In order address the question of disordered networks, we now have added new Fig. 5 that shows the effect of changed connectivity, including holes and longer spectrin connections. Both for Fig. 5 and in the introduction, we now give a more in-depth discussion of the role of inhomogeneities in the actin-spectrin network.

3. Nonmuscle myosinIIA (NMIIA) is conspicuously missing from the model. See https://www.pnas.org/content/115/19/E4377.short. The authors should comment on the role played by NMIIA potentially.

The same question was raised by reviewer #2. Please refer to the corresponding answer above.

4. Finally there is only one result with respect to malaria in Figure 6 and even there it is not clear what the different lines in Figures E and F are (legend has only one value). But it is clear that these trends don't match very well with Figure 6B and C and further explanation is needed as to why that may be.

We first note that our whole work is focused on the effect of malaria-infections. It is true however that Fig. 6 (now Fig. 7) is the only one which shows a direct comparison with experiments and that this comparison can only be qualitative because we are forced to simulate on a smaller scale than what is monitored in experiments. The different lines are color-coded and represent different values of binding energy between KAHRP and actin, as shown by the legend. The qualitative agreement means that changes in affinity can explain the experimentally observed relocalization, which is not trivial considering the different ways in which KAHRP could cluster in the cytoskeleton. The reason why the simulation data has more structure is that it is less averaged than in the experiments. In particular, this explains why we see a sharper profile around the peak (anti-correlation in E and second peak in H) and peaks at shorter distances in (E). A similar effect can also be observed in the Pan-paper, which uses the same methodology.

For Fig. 7E we now have added a reference to the color bar. The comparison between experimental and simulated correlations is discussed around line 503. In line 611, we explain that the Pan-work shows the same characteristics. In the discussion (lines 551-554), we now clarify that a complete simulation is not possible at the current stage due to missing information, but that we focus on potential mechanisms underlying the experimentally observed changes to actin- and KAHRP-localizations.
